# Approaches for Predicting the Occurrence of Challenging Behaviors in Individuals with Autism Spectrum Disorder: A Narrative Review

**DOI:** 10.3390/jpm15100453

**Published:** 2025-09-29

**Authors:** Jennifer Ferina, Emma Dando, Conor Anderson, Jenny Foster, Johanna Lantz, Theresa Hamlin, Juergen Hahn

**Affiliations:** 1 Department of Biomedical Engineering, Rensselaer Polytechnic Institute, Troy, NY 12180, USA; jen24fer@gmail.com (J.F.); dandoe@rpi.edu (E.D.); 2 Center for Biotechnology and Interdisciplinary Studies, Rensselaer Polytechnic Institute, Troy, NY 12180, USA; 3 The Center for Discovery, Harris, NY 12742, USA; canderson@tcfd.org (C.A.); jenny.foster@tcfd.org (J.F.); jlantz@tcfd.org (J.L.); thamlin@tcfd.org (T.H.); 4 Department of Chemical and Biological Engineering, Rensselaer Polytechnic Institute, Troy, NY 12180, USA

**Keywords:** autism spectrum disorder, behavior prediction, challenging behavior, machine learning

## Abstract

The presentation of autism spectrum disorder can be highly heterogeneous, and a significant percentage of individuals with autism exhibit challenging behaviors. However, the frequency and severity of challenging behaviors can vary significantly over time, even for the same person, and it is often unclear what triggers a behavior. Being able to predict the occurrence of challenging behaviors has significant potential for improving the safety of individuals with ASD, as well as quality of life for them and their caregivers. Given the large heterogeneity found in the presentation of autism, most predictions need to be personalized to the individual or at least a subgroup of individuals. This work reviews the state of research in the field for predicting behaviors, ranging from short-term predictions just a few moments before a behavior occurs to predicting the probability of challenging behaviors a day in advance. Several reviewed approaches provide promising initial results, but few studies have been conducted on populations large and diverse enough to determine their generalizability.

## 1. ASD and Challenging Behaviors

The prevalence of autism spectrum disorder (ASD) continues to increase [1]; the latest CDC surveillance study of children between the ages of four and eight found that 1 in 31 children are diagnosed with ASD [2]. The presentation of ASD is highly heterogeneous, as are the needs of individuals with ASD [3]. Many individuals with ASD exhibit challenging behaviors, which can include disruptive behavior, aggressive behavior, self-injurious behavior (SIB), and elopement, among others. There are several consequences to these behaviors: aggressive behavior and SIB, which can cause physical danger to the individual or others; elopement, which can endanger the individual if they enter a dangerous environment; and disruptive behavior, a common challenging behavior that makes it difficult for an individual to function in a classroom or society. Behavior specialists focus on reducing and replacing these challenging behaviors by determining their function. However, individuals with severe behavior problems often are non- or minimally speaking, and it may be difficult to understand why the behaviors are occurring.

The relationship between age and the frequency and severity of challenging behaviors is still unclear. A study of children aged three to fourteen did not show any significant differences in individual, parent-reported challenging behaviors between different age groups; however, there was a nearly significant decrease in challenging behaviors overall [4]. A systematic review and meta-analysis of longitudinal studies showed significance of a similar, slight decrease in challenging behaviors over time [5]. However, this does not demonstrate a clear correlation with age, as the reduction in behaviors over time was not influenced by the age of the participants when the study began; instead, it was influenced by the duration of the study and how old the participants were when it ended [5]. This may indicate a weaker or more complicated relationship with age. A potential complication of this relationship is that individual challenging behaviors may have different age progressions. A small study that interviewed twelve parents of children with ASD about their experiences with puberty reported that some challenging behaviors, i.e., self-harm, decreased, while others, namely aggression and inappropriate sexual behaviors, increased [6]. Another study provided a more granular exploration, looking at specific self-harm behaviors from the ages of three to nineteen [7]. They found that only five of the eleven behaviors examined had a significant relationship with age, with some showing periods of elevation and others more linear increases [7]. It has been theorized that the relationship between challenging behaviors and age may be influenced by ASD severity and co-occurring medical conditions [8].

Challenging behaviors can be psychological or physiological in origin and may be due to a unique combination for each individual. A recent area of focus for the origin of challenging behaviors has been co-occurring conditions (COCs) [9]. COCs are more common for individuals with ASD than those without [10,11], and children with ASD make up 35–40% of those with severe or chronic medical conditions [11]. Frequently reported types of COCs include gastrointestinal (GI) problems, epilepsy, sleep disorders, and immune conditions [12,13], though other types, such as psychological conditions, are also of interest [13]. Incorporating COCs into analyses and predictions of challenging behavior has the potential to be a quantifiable and repeatable way to take into account variations between individuals and tailor predictions.

As the prediction of challenging behaviors is a reasonably new topic, there is currently no complete review of the state of research. A conference paper in 2022 outlined a potential methodology and preliminary research for a similar review, though it was exclusively about wearable sensors and their feasibility, and, interestingly, no review was actually conducted [14]. This work addresses this shortcoming in the field and presents an overview of the literature on challenging behavior prediction.

## 2. Types and Prevalence of Challenging Behaviors

There are several clinically relevant challenging behaviors. In Table 1, four behavior types are defined, and examples are provided. Severity is not discussed, as it is rarely reported, and when it is, methodologies vary. When discussing SIBs, this review focuses on non-suicidal SIBs [7] and includes some behaviors defined as self-stimulatory and stereotypical motor movements (SMMs), such as head banging.

The prevalence of challenging behaviors can change between studies, potentially due to possible variations in how clinicians define behaviors, numbers are reported, and data are collected [16]. Aggression and SIB prevalence were tracked in the Autism and Developmental Disabilities Monitoring (ADDM) Network, a population surveillance of over 200,000 eight-year-olds with ASD [15]. A 2023 review found a similar prevalence of aggression and SIB in other studies [5], but a meta-analysis noted in the same review showed a higher prevalence, 42%, of SIB [5,16]. Elopement has not been studied in as large a population sample, which may explain the large variance in prevalence. A Center for Disease Control (CDC) study of 1218 families reported a prevalence of 49.1% [18,19], but a smaller follow-up study reported a prevalence of only 32.7–34.6%, depending on the presence of co-occurring intellectual disability (ID) [17]. Disruptive behavior is typically reported as oppositional defiant disorder (ODD), conduct disorder, or both, though this behavior is not limited to individuals with these disorders. Additionally, some studies focus on one aspect of this category. A 2008 study on ODD found a prevalence of 28.1% [20]. A 2023 meta-analysis that used the label disruptive behavior reported a pooled prevalence of 28%, but individual studies ranged from 21% to 36% [13]. It is clear that these behaviors are a pressing concern, as they may affect between a quarter and half of children with ASD and can impair their ability to function, potentially requiring them to stay in a residential facility or receive around-the-clock care [5,21].

### Behavior Frequency and Co-Occurrence

An individual may have none, one, or several challenging behaviors. A large population study of 2116 individuals with ASD examined challenging behavior types using k-means clustering, finding that behaviors typically appear in their own cluster, but one cluster showed co-occurrence of aggression, elopement, stereotypy, noncompliance, and tantrums [22]. Another study found that aggression and stereotypy tended to co-occur with SIB; however, they also found that COCs did not predict if an individual had SIB [23]. A meta-analysis explored how challenging behavior changed over time, which found a small but significant decrease in challenging behavior [5]. Examining trends in challenging behavior, including which behaviors they tend to co-occur with, is of interest for behavior prediction and is used by some groups for challenging behavior prediction. The next section will examine common COCs with ASD and the complex relationship COCs can have with each other and challenging behaviors.

## 3. Co-Occurring Conditions and ASD

The presentation of co-occurring conditions associated with ASD are complex and reduce quality of life. In a large study of individuals with ASD and their typically developing (TD) siblings, 74% of individuals with ASD had at least one COC and had, on average, more COCs than their TD counterparts [10]. An analysis of national surveys from 2021–2022 found that 59.28% of children with ASD, as opposed to 2.6% without ASD, had multiple COCs or chronic conditions [11]. All of these COCs have the potential to cause pain or discomfort, and some tend to co-occur with each other. For example, Vargason et al. found three different COC clusters when examining insurance claims: two clusters with high and low prevalence of COCs, and one cluster with a high prevalence of developmental delays [24]. A more recent study, focused on COCs and challenging behaviors in ASD, also revealed three COC clusters: one with allergies/sinus infections and non-asthma respiratory illness; one with gastroesophageal reflux disease (GERD), constipation, and epilepsy; and one with headache/migraine and sleep dysregulation [25]. These studies indicate possible ASD subtypes; thus, it may be prudent to examine individuals for other COCs as other possible sources of pain or discomfort when one COC is identified. This may be especially valuable for individuals with language impairments, as challenging behaviors may be the only indication of an underlying medical issue [9]. Additionally, it may be highly relevant for behavior prediction models to incorporate COCs, which may be individual predictors, as shown in Table 2.

### 3.1. Gastrointestinal Conditions

GI disorders and symptoms are widely reported in the literature due to their prevalence and interference with daily life. The exact prevalence is difficult to determine, as reports vary significantly, from 9% to 91% [9]. This range is presumed to be due to methodological variations, namely how populations are recruited and GI issues are recorded and defined [29]. An all-ages meta-study of global prevalence, which combined diagnoses and symptoms, had a prevalence of 48.7% [30]. In contrast, another all-ages meta-analysis divided GI conditions into disorders, which affected 21% of those with ASD, and problems, which affected 39% [13]. Regardless of the exact percentages, it has been well established that GI issues are more common for those with ASD than without [9,29]. Additionally, a study of children 2–3.5 years old found that those with ASD were also more likely to experience multiple GI symptoms than those without [29].

Currently, there is no defined mechanism or pathway that links GI conditions and ASD [9,12,29]. It has been theorized that there may not be a single, ubiquitous link between these conditions; instead, there may be different subtypes of how ASD and GI conditions interact, with each subtype involving one or more mechanisms [12]. The core of this research is the communication pathways between the digestive track and neurological system, termed the gut–brain axis [9]. A predominant theory is that individuals with ASD have “leaky guts”—that they absorb excess toxins that are generated as a by-product of or typically disposed of during the digestive process—due to abnormally permeable GI tracts [12,31]. It has also been suggested that mucosal cell dysfunction may increase inflammation, potentially contributing to GI issues [12] and ASD [9]. Other theories focus on genetic alterations, such as of *MET* [9,12] and *SLC6A4* [9]. Alterations to MET receptor tyrosine kinase have been associated with GI dysfunction in individuals with ASD [9,12]. *SLC6A4* encodes the serotonin reuptake transporter, and serotonin variations have been linked to slow gut activity [9,12,31], mood issues such as anxiety, and cognition issues [12].

GI differences in individuals with ASD can also be observed in the microbiome [9,32]. One study shows that individuals with ASD have fewer species of *Bifidobacter* and more species of *Lactobacillus* than TD children [32]. However, other studies have contradicted these results, instead showing greater microbiome diversity [12]. This may indicate that the microbiome’s influence is more complicated than simply more or less diverse [12], or it is non-ubiquitous. One theory is that alterations in the microbiome affect inflammation levels and correspondingly the immune response, which may impact ASD [9]. When using microbiome transfer from a healthy donor, GI and ASD symptoms improved, including abberrant behaviors [33]. Several studies also show differences between the fecal metabolite levels in ASD versus TD children [34,35,36]. In particular, there is lower microbial diversity [34], elevated isopropanol and p-cresol, and different neurotransmitter levels [34].

ASD is associated with higher sensory sensitivity, potentially limiting an individual’s diet; 84% of children with ASD were found to exhibit food selectivity [37]. While food intake may not be significantly associated with GI symptoms [38], there are some feeding-associated problems, including rapid eating, food stealing, and food refusal, that were associated with higher rates of GI symptoms, challenging behaviors, and sensory sensitivity [37]. Higher rates of GI symptoms are also associated with ASD severity [32]. Children with ASD and GI problems also have an increased allergy prevalence [33]; which may contribute to food restrictions and GI symptoms.

Individuals with ASD are treated with a wide range of medications with varying side effect profiles which, in some cases, may be responsible for an individual’s symptoms [31]. Many common medications may cause GI symptoms including changes in appetite, diarrhea, constipation, nausea, and vomiting [31,39]. Certain classes of medication are more frequently associated with certain side effects; for example, diarrhea is a common side effect of SSRIs [39] and antipsychotics [31].

#### Associations with Behavior

GI symptoms, in particular nausea, abdominal pain, and constipation, were shown to be associated with affective problems, or mood disorders, in children with ASD [40]. Challenging behaviors were also associated with affective problems [40]. Anxiety, in particular, has been theorized to have a bidirectional relationship with GI issues [12]. In preschool-aged children, aggressive behavior was significantly predictive of nausea [38]. GERD was found to be associated with aggression and SIB [25]. Interestingly, these correlations may not be limited to those with ASD. One study found that aggression, restricted stereotyped behaviors, attention difficulties, and sleep issues were all more common in children with co-occurring GI symptoms, regardless of whether they had been diagnosed with ASD [29]. Additionally, the study reported higher instances of SIB and sleep issues if a child with ASD had multiple co-occurring GI symptoms [29]. Treatment for GI symptoms has been shown to reduce behaviors such as agitation and screaming [9].

Negative mealtime behaviors were also associated with sleep disorders and emphasized the correlation between sleep problems and aggression [41].

### 3.2. Epilepsy

The prevalence of co-occurring epilepsy with ASD is typically cited as between approximately 25% and 30% in adults [12], though estimates can range significantly. In part, this is expected to be based on age and sampled population. An epidemiological study of children aged 3–12 in Shanghai, China found that 11.5% of children with ASD had epilepsy, compared to only 0.2% of those without [26], while a study of the Medicaid records of adults found that 22.7% of those with ASD had epilepsy, compared to 4.8% without [42].

The biological relationship between ASD and epilepsy is still unclear, in part due to the complex origins of both conditions [12]. The relationship between ASD and epilepsy subtypes varies between studies, further complicating research [12]. A study using the SPARK database found similar relationships between COCs, regardless of whether an individual had ASD or not. The authors suggested that this may indicate that ASD does not impact the etiology of epilepsy [10]. Current theories on the connection between ASD and epilepsy encompass every level of the brain. Some studies suggested that abnormal connectivity between different regions of the brain [26] or atypical synaptic plasticity may be responsible [12]. Another suggested that seizures could cause damage that might contribute to ASD [26]. Beyond the brain, it has also been suggested that the link may be genetic in origin, as a number of genes have been reported to be involved in both conditions [12,26]. Chromosomal defects can also cause genetic differences and have been associated with ASD and epilepsy [12]. Another review suggested that the relationship between ASD and epilepsy may be related to inflammation and the immune system [9].

#### Associations with Behavior

Epilepsy has been associated with several social and behavioral symptoms for those with ASD [12]. Various studies have shown more profound ASD symptoms, such as increased repetitive object use; increased sensory focus; and difficulties with eye contact, facial recognition, and other aspects of socialization [12]. A study, noted in the same review, of 2645 children with ASD demonstrated a statistically significant increase in self-injurious, compulsive, and sameness behaviors among children with co-occurring epilepsy, even when adjusted for age and sex [12,43]. Some symptoms of ASD have shown improvement when co-occurring epilepsy could be treated [12]. Additionally, those with ASD and epilepsy have an increased risk of co-occurring ID [12,26].

In addition to the direct effects of epilepsy on behavior, the medications used to treat epilepsy can affect behavior [39,44]. While many epilepsy medications are prescribed to reduce irritability and aggressive behavior in individuals with ASD, in some cases, they can instead increase irritability and aggression [39]. These adverse side effects are more common for certain medications, such as levetiracetam and perampanel, and they may be more common in those with a psychiatric or behavioral history [44].

### 3.3. Sleep Disorders

Problematic sleep is a known concern for individuals with ASD; those with ASD have a pooled prevalence of 13% of sleep disorders compared to only 3.7% in the TD population [27]. However, other reviews have reported rates between 40% and 80% [9] compared to between 9% and 50% in children without ASD [12]. The variation between reports may be due to methodological variations in recruitment and how sleep problems were defined and recorded. Compared to TD children, children with ASD had reduced sleep duration, increased sleep onset latency, and longer time awake before sleep onset, in addition to decreased REM sleep and more stage 1 sleep using objective measures [45]. Subjective measures of sleep onset delay and latency, sleep anxiety, bedtime resistance, restorative value of sleep, sleep-disordered breathing, daytime awakenings, night awakenings, parasomnias, and sleep problems were higher in children with ASD, while sleep efficiency was lower [45]. There is some evidence that sleep problems change for people with ASD as they age [12].

Several theories have been proposed to explain the increase in sleep disorders among individuals with ASD. Sleep is psychological as well as physiological, so behavioral issues such as difficulty changing activities, common in ASD, may impact sleep [12]. Additionally, sensory issues, either heightened to stimuli or reduced due to environmental cues, can impact the psychological state and circadian rhythm [12]. Physiologically, melatonin levels, which regulate the circadian rhythm, have been reported to be altered in individuals with ASD [46]. Often they are reported as lower [12,46], though they have also been reported to be higher during the day and lowered at night compared to controls, or to have delayed rhythms [46]. The origin of these alterations is unknown, but based on genetic studies, which have shown no evidence of mutations or dysfunctions in melatonin receptors, a prominent theory is that synthesis is altered in individuals with ASD [46]. The melatonin synthesis pathway can be traced to tryptophan, which is a precursor to serotonin, then melatonin, among other pathways [47]. The gut microbiome affects the equilibrium between these pathways, with serotonin synthesis using only a small portion of tryptophan, so even small variations have significant consequences [47]. Over 90% of serotonin is synthesized in the GI system, but even serotonin synthesized in the nervous system may be affected by the gut microbiome [47]. Individuals with ASD typically have higher and more variable levels of serotonin, which is the opposite of what is found for melatonin. Some studies indicate that the conversion of serotonin to melatonin may be where this pathway is altered for individuals with ASD [46]. Genetic links may also impact the relationship between sleep and ASD, as some genes that have been implicated in sleep and circadian rhythms may be altered in individuals with ASD [12]. There may also be a bidirectional link between sleep and inflammation in individuals with ASD. Increased levels of inflammation have been reported in individuals with ASD, including pro-inflammatory cytokines in the brain, which are associated with sleep issues [9]. Conversely, mouse studies indicate that sleep issues may increase inflammation in the colon [9].

#### Associations with Behavior

Sleep and affective problems are associated in children with ASD [40]. A small study (n = 3) identified fatigue as the setting event, or an event increasing the likelihood of the behavior, for problem behavior [48]. Associations have also been found between sleep dysregulation/disturbances, aggression, and SIB [25,41,49]. Interestingly, the behavioral association appears to change depending on the sleep issue [12]. For example, decreased sleep and parasomnia have been associated with worsened repetitive behaviors and communication, but reduced sleep has also been associated with over-adherence to rituals or routines, SIB, aggression, and tantrums [12]. The link between behavior and sleep may also alter with age, as one study found a higher-than-expected relationship between sleep issues and disruptive behavior for children under six, but not over [50].

### 3.4. Allergies and Immune Conditions

According to a National Health Interview Survey, children with ASD were 1.82 times more likely to have an allergic condition than a TD child [28]. Of the allergies tested, respiratory allergies were the most common—18.73% in children with ASD versus 12.08% without—although food and skin allergies also had a significantly higher prevalence [28].

The prevalence of other immunological COCs has also been investigated, though it is complicated by studies suggesting that immune alterations may be a core aspect of ASD [9]. A retrospective analysis of medical records noted that individuals with ASD are significantly more likely to have had infections, 21.5% versus 17.4% [42]. Allergic rhinitis has also been reported to be more common among individuals with ASD [51], as has psoriasis [12]. There have been mixed reports of asthma [9,12] and autoimmune conditions [52,53] co-occurring with ASD.

#### Associations with Behavior

The relationship between immune conditions and challenging behaviors is still unclear. Connections between allergy type/presence and challenging behaviors have not been explored in the literature. Increased levels of inflammation have been related to ASD symptom severity, while decreased transcription growth factor beta 1 levels have been associated with behavior and adaptive functioning problems [12]. Treatments designed to modulate the immune system have had mixed results [9,12], though atypical antipsychotics, commonly used for individuals with ASD, have been reported to have some anti-inflammatory effects [9]. While immunological conditions have not been directly associated with challenging behaviors, several studies have theorized that they are part of feedback loops with other COCs, which contribute to challenging behaviors [9,12].

### 3.5. Menses

Menses can cause discomfort, so they should be examined as a potential contributor to challenging behaviors. Few studies have focused on menstrual issues, and those that did predominantly involved individuals less severely affected by ASD [54,55,56]. Individuals with ASD had more menstrual symptoms than those without, with the majority of complaints being emotional or sensory [54]. A study of 1230 women, 361 of whom had ASD, reported that women with ASD were more likely to have reproductive COCs, more menstrual symptoms, irregular onset of puberty, and irregular period lengths [57].

#### Associations with Behavior

Reports from individuals with ASD have indicated increased emotional regulation issues [55,56] and meltdowns [56] during their periods. A small study of three individuals showed that treating menstrual symptoms, in addition to task demands, reduced challenging behaviors [58].

## 4. Profound ASD and Challenging Behaviors

Profound ASD represents 26.7% of the ASD population and is characterized by being non- or minimally verbal/speaking or having an IQ < 50 [15]. Often, this population requires full-time support and care. This group is often under-served in the literature [3,59,60,61], to the point where the CDC now reports profound ASD in their prevalence studies. Although ASD is more common in boys than girls, profound ASD has a higher prevalence in girls [15]. Those with profound ASD have a higher prevalence of SIB—36.5% versus 24.9% of those with non-profound ASD—and both populations exhibit approximately 50% prevalence of aggressive behaviors [15]. However, the *frequency* of challenging behaviors has not been found to be significantly associated with traits on the Autism Diagnostic Observation Schedule-2, a measure of levels of autistic traits, or the child’s developmental level on the Mullen Early Learning Composite [62].

### Psychological Functions of Behavior

Although this review is focused on physiological reasons for challenging behaviors, these behaviors may also be psychologically motivated. Behaviors are typically separated into two functions in the psychological field: automatic (independent of the social environment) or social reinforcement [63]. Most frequently, behaviors function as a means of escape [64,65].

Negative affectivity, the tendency to experience negative emotions, in young children was found to be significantly associated with more frequent challenging behaviors at follow-up [62]. The main limitation of this field is that it usually examines very small sample sizes in specific settings. Additionally, studies are often performed on individuals with ID, but not necessarily co-occurring ASD. Challenging behaviors are often determined to be performed for multiple different functions in this field [66,67,68,69]. Thus, behavior prediction may be more successful and translatable to a clinical setting when using common factors that can be physiologically and quantitatively measured for larger populations.

## 5. State of Behavior Prediction Research

While many studies examine the association between certain physiological and psychological features and the presence, absence, or frequency of behavior, and this information is quite useful for preparing caregivers for children who will potentially develop these behaviors, these analyses only report one time point. The daily care experience for these individuals and caregivers is very different; days with challenging behavior can prevent plans such as medical appointments, and the caregiver or individual could potentially be injured from the challenging behavior. Thus, predicting when these behaviors will occur is of great interest to the individuals who experience them, and their caregivers.

Current reviews of behavior prediction focus mainly on wearable sensors [14,70,71,72,73,74,75,76]. However, often, these reviews have a scope that does not include challenging behavior prediction, for example, emotion recognition [77], or ASD diagnosis [78]. A recent perspective highlighted the literature gap that exists regarding prediction and intervention for challenging behaviors for children with ASD, particularly in their natural environments [79]. This literature review highlights wearable sensors, non-wearable sensors, and approaches based on manually recorded data as techniques for challenging behavior prediction in ASD. This review also includes behavior and mood/stress detection, as it does involve detecting or sensing the behavior or related precursor when it occurs, like most sensor approaches that predict challenging behaviors.

Due to the emerging nature of this field, many studies suffer from limitations, most notably limited samples. The majority of studies used small sample sizes. On average, approaches that used bed and movement sensors had the smallest sample sizes, although only five reviewed studies of any methodology had a sample size larger than twenty-five, not including studies that used databases. There are some studies that have thus far only examined neurotypical (NT) or TD populations [80,81,82,83], and one looked at children with ID [84]. The majority of studies also focused on children, which limits the translatability to adult populations. Women are underrepresented, and racial and ethnic demographics are frequently unreported. Most studies take place in controlled environments and have not been validated in real-world scenarios. Nearly half of the studies reviewed are conference papers or preprints. Future work will be required to validate the results presented here and to determine if they can be translated to larger, more diverse populations and real-life scenarios. Work on behavioral detection is also presented here, as it is presumed to be a relevant component to eventual behavioral prediction. However, studies that have attempted to translate behavior detection methods to prediction have seen notable decreases in accuracy, highlighting a need for future work.

### 5.1. Wearable Sensor Approaches

A recent commentary by Gifford and Valdovinos notes that while sensor results are promising, they have yet to be applied clinically [85]. Functional behavior analysis (FBA) is still the gold standard for challenging behavior treatment and intervention, and the current limitation of wearables is the lack of an instant alert when a challenging behavior is occurring [85]. The authors note the promise of sensor applications in challenging behavior as a tool to complement the much more established field of FBA; for example, observing environmental events that an individual experiences when the sensor signal begins to change could help to identify the trigger for challenging behavior, especially in cases where it is difficult to otherwise identify [85].

#### 5.1.1. Physiological Signals

Studies that use physiological signals are listed in Table 3. One of the first approaches using physiological approaches to detect children’s affective states was performed in 2008, using several physiological signals. Electrocardiogram (ECG), electrodermal analysis (EDA), electromyography (EMG), and skin temperature recordings were feature-engineered, fed into individual support vector machines (SVMs), and predicted anxiety with 79.5% accuracy, liking with 85%, and engagement with 84.3% [86]. While encouraging, the model did not directly predict challenging behaviors, and it was a small study of six individuals [86]. A similar study by Sarabadini et al. of 15 children with ASD used ECG, skin conductance, skin temperature, and respiration measures to predict affective states [87]. They applied five different classifiers and found that the best performance, an average accuracy of 78.1% at differentiating positive and negative high-arousal states, was obtained from a majority vote of the classifiers [87].

Laarhoven and coworkers used a wearable device that measured and categorized respiration patterns, in a small study (n = 5) to examine respiration patterns during challenging behaviors [88]. However, only two individuals had a relationship between behaviors and breathing patterns [88]. This study highlighted that behaviors are highly individual and that while biosensors have potential to record behaviors, behaviors may occur for reasons not recorded by the biosensor [88]. Another study by Ferguson and colleagues found a similar issue when correlating EDA and challenging behavior [89]. In a study of eight adolescents with ASD, correlations between a change in EDA and challenging behavior were only found 60% of the time, and EDA only returned to baseline after a behavior 45% of the time [89].

Several preliminary studies examined the correlation between heart rate (HR) and challenging behaviors, either to predict them directly or to better understand potential relationships that could be used to predict challenging behaviors in the future. First done by Freeman et al. with an FBA approach in 1999 [90], two men with developmental disabilities wore ECG sensors to detect HR, and HR was compared with when behaviors occurred [90]. Though this study could not be generalized with such a small sample size, the authors found that HR increased for both men after challenging behaviors [90]. A subsequent study on one individual applied the Learning from Examples using Rough Sets (LERS) algorithm to the data, including HR and environmental data, such as staff praise. The algorithm was able to produce rules to predict under what conditions certain types of behavior were likely to occur, although no evaluation metrics were presented [91]. One study of a boy with ASD showed significant associations between different activity types (high or low arousal activities) and HR [92]. A study of three individuals with ASD and varying levels of ID examined how individuals’ HR, detected using a band around the torso and transmitted to a wristwatch, changed during challenging behavior [93]. For SIB, only one participant showed a significant difference [93]. A study of preschoolers with ASD (n = 13) found that baseline-corrected HR could predict challenging behavior with an area under the receiver operator curve (AUROC) of 0.72 [94], but that heart rate variability (HRV) was not a significant predictor.

In the same year, Masino and colleagues used ECGs to predict stress that may precede challenging behaviors [95]. They showed 22 children with ASD a relaxing video to record baseline HR and beat-to-beat parameters, then gave them each two frustrating tasks [95]. Masino et al. were able to differentiate between stressed and relaxed states with an area under the curve (AUC) of 91% using radial basis function (RBF) kernel SVM [95]. A similar study used a wearable that measured HR and detected whether it reached a certain threshold to alert caregivers to stress in children with ASD; however, the reason was unidentifiable or a false alarm for over a third of the alerts [96].

A study by Khular and colleagues used a wristband to measure HR, galvanic skin response, and skin temperature; preprocessed the data using isolation forest outlier detection for initial classification; and sent the data to a deep learning model to predict meltdowns [97]. They tried both approaches individually but found that a hybrid convolutional neural network (CNN) long-short term memory (LSTM) (CNN–LSTM) network performed the best, with 96% accuracy [97]. Interestingly, the model performed similarly for the ten children with ASD and the five TD children, indicating that detecting meltdowns using these parameters can be extended to multiple populations [97].

ECG and HR signals have also been used to detect like or dislike valence states in children with ASD [98], finding that ensemble classifiers performed the best—84.7% on the control group and 81% in children with ASD—when using db8 HRV. Interestingly, HRV alone required derived statistical features to separate the valence states [98]. The most important features were ECG signal features [98].

#### 5.1.2. Movement Sensors

Movement sensors, such as accelerometers and gyroscopes, have been used by several groups to detect and predict challenging behaviors and behavior groups, which can include challenging behaviors, like SMMs, listed in Table 4. Goodwin and colleagues performed small studies of six individuals to detect SMMs using accelerometers, with features fed into a C4.5 classifier, finding an average of over 90% accuracy [99,100]. Wrist and torso accelerometers were also used to detect SMMs in six individuals with ASD, which were predicted using a decision tree (DT) and linear SVM, with the SVM outperforming the DT with a range of accuracies from 81.2% to 99.1% across individuals and experiments [101]. Rad and coworkers extended the work of the Goodwin group by applying CNNs to accelerometer data in a study of six children to detect SMMs [102]. The CNN, especially when augmented with transfer learning, outperformed the SVM classifier [102]. In particular, features learned by the CNN were more predictive than manually extracted features, achieving an F1 score of 0.75 for one experiment and 0.48 for the other, outperforming the Goodwin studies [102]. Gesture recognition in children with ASD has also been performed using accelerometer and gyroscope wrist sensors; Siddiqui et al. conducted a study of 24 gestures of ten children with ASD and employed several ML approaches to detect each gesture [103]. An accuracy of approximately 91% was achieved with random forest (RF), neural network (NN), and k-nearest neighbors (KNN) [103].

Wrist and ankle straps with accelerometer sensors were also used to detect aggression, SIB, and disruption [104]. Three classifiers were trained to predict staff-simulated challenging behaviors, including naive bayes, DT, and SVM, tested on an Activities of Daily Living dataset without challenging behaviors, and finally validated on a child with ASD and challenging behavior; SVM produced the highest accuracy of 69.7% [104]. Notably, the child did not tolerate ankle sensors [104]. Min and coworkers conducted a study to determine optimal sensor placement for two children with ASD [105]. They tested accelerometer sensors worn on the wrists and back to detect body-rocking and hand-flapping. While wrist sensors outperformed the back sensor for hand-flapping, they found that the back sensor was able to detect both behaviors with reasonable accuracy [105]. Another accelerometer study, tested on self-stimulatory behaviors of four children with ASD, two of whom exhibited different forms of SIB, used linear predictive coding as an autoregressive model; they achieved an average accuracy of 92.7% for predicting self-stimulatory behaviors [106]. Min and coworkers performed a later study using hidden markov models (HMMs) to detect self-stimulatory behavior patterns and achieved a detection rate of 91.5% across four individuals [107]. They combined the accelerometer sensor with video recording and saved the corresponding videos when the behavior events were detected from the accelerometer sensor data [107]. An earlier study by Westeyn et al. in 2005 used HMMs to detect self-stimulatory behavior, although it was only tested on a NT adult wearing accelerometers and mimicking the behaviors [80]. They employed a wrist sensor, a waist sensor, and an ankle sensor, and they tried both isolated recognition and continuous recognition of the behaviors [80]. With isolated recognition, their HMM approach achieved 90.5% accuracy; with continuous recognition, it achieved 92.86% [80].

Cantin-Garside et al. used variable numbers of accelerometers on eleven participants to detect SIB, focusing on nonlinear motor variability in addition to the time and frequency features that were typically collected with accelerometer data. They preprocessed the data by performing LASSO and principal component analysis (PCA) for feature extraction, then they used a multilevel logistic regression (LR) model to predict SIB with an accuracy of 74.7% [108]. That same year, Cantin-Garside et al. used a similar methodology, a variable number of accelerometers on eleven participants, to detect the presence and type of SIB [109]. For binary SIB detection, KNN achieved the highest average accuracy of 93.0%, and SVM achieved 92.2% [109]. The 24 individual types of SIB had variable classification accuracies, with head banging, finger picking, and kicking being among the most detectable by KNN [109].

A team of researchers also tested putting accelerometers in toys to detect aggression, with accelerometer data sent to a recurrent neural network (RNN) [81]. The set up was that the toys detect aggressive behavior and send the information to a social robot, which instructs the child to stop [110]. The framework has not yet been experimentally evaluated on children with ASD; however, the behaviors were predicted successfully with NT adults and children [81].

#### 5.1.3. Combined Analysis

Wearables, such as smartwatches, measuring EDA, pulse, and its variability, have been successfully used for behavior prediction [111], as have approaches combining them with accelerometer data, shown in Table 5. Goodwin and colleagues used three minutes of prior physiological and accelerometer data collected with a wristband sensor to predict aggression for 20 youth with ASD one minute before it occurred using ridge-regularized LR [111]. They found that individual models produced a higher average AUC, 0.84, than population models, 0.71 [111]. They used features of EDA, inter-beat interval (IBI) and blood volume pulse (BVP), as well as acceleration in all directions [111]. Alban and colleagues used wristband sensors to predict challenging behaviors for five children with ASD; the features used included acceleration, EDA, skin temperature, HR, and BVP [112]. They applied several machine learning methods to predict challenging behaviors from these recordings, including SVM, multilayer perceptron (MLP), DT, and extreme gradient boosting (XGBoost); XGBoost achieved the best performance in prediction accuracy, 99%, and runtime [112]. Notably, the physiological features, particularly HR, were the most important for predicting challenging behaviors [112].

EDA was also used in conjunction with BVP and an accelerometer to predict aggression, which included SIB and emotional dysregulation, in two recent studies by Imbiriba and colleagues [113,114]. They initially performed a study with 20 children to detect aggression, achieving up to 98% accuracy for both individual and population models using PCA followed by an RBF kernel SVM [113]. In a study across four inpatient hospitals of 70 children with ASD, Imbiriba and colleagues used wrist-worn biosensors to predict aggression three minutes before it occurred, augmented these physiological features with the most recent behavior data, and achieved a mean AUROC of 80% [114]. The best performance was produced by LR [114].

A recent conference paper of a small study (n = 5) utilized a wearable sensor to detect challenging behaviors that arise before a meltdown [115]. Acceleration data, EDA, temperature, HR, and BVP were collected by the wearable and then fed to the compared models, DT, SVM, MLP, and XGBoost [115]. XGBoost performed the best of the models with an accuracy of 91% and F1 score of 88%. The three most important signals were, in descending order, HR, EDA, and temperature [115].

A framework proposed by Al Banna et al. included a wearable device, camera, and toys with radio frequency identification (RFID) [82]. The proposed wearable would have an accelerometer, gyroscope, magnetometer, GPS, pedometer, HR, and temperature sensors [82]. A camera would take photos every five minutes to assess the individual’s emotions [82]. The proposed framework was meant for use during the COVID-19 pandemic to allow caregivers to monitor the child when quarantined, and it also included learning and calming prompts through the wearable [82]. However, only the CNN transfer learning model to recognize emotion from images was tested [82].

Another interesting approach by Zheng and coworkers used multimodal methods to predict precursors to challenging behaviors in seven children with ASD [116]. Using a Microsoft Kinect device, along with a wearable wristband and sensor hoodie, they were able to collect data about facial expressions and head rotations, acceleration, BVP, EDA, and temperature [116]. A therapist simulated conditions that would typically induce challenging behaviors for the child, and behavioral states were recorded. The researchers then used several different ML algorithms to predict precursors to challenging behavior: for the population model, RF and NN were the best-performing, and for individual models, RF was the best performing [116]. Individual models showed high accuracy of 98.51%, though population models only produced 82.36% [116]. This is of interest, as it shows how individualized behavior predictors are. The model could allow up to 90 s of warning before the precursor events occurred without a substantial decrease in performance [116]. Although the delay between the precursor event and the challenging behavior varies and is unknown, the caregiver receives at least 90 s of warning before the problem behavior occurs [116]. Another approach with clothing sensors was designed by Zwillig and coworkers to predict challenging behavior; however, results have not yet been reported for this clinical trial [117].

Goncalves and coworkers compared wearable and non-wearable sensor approaches for classifying hand-flapping stereotypy: an accelerometer watch and a Microsoft Kinect sensor [118]. The accelerometer had a higher detection rate of 76% than the Kinect, which had only 51%, but the authors noted the limitation of the field of view of the Kinect sensor compared to the accelerometer [118].

Accelerometers were the most effective in a comparative study performed on eleven boys to determine the most effective wearable sensor, between EDA, three-lead accelerometer, and skin temperature monitoring, to detect and predict challenging behaviors in a classroom rather than a laboratory setting [119]. The accelerometer performed the best, with an AUROC of 0.710, compared to 0.524 for EDA and 0.629 for skin temperature [119]. Interestingly, the general population model often provided better predictions than patient-specific models [119]. The study also noted the specific goal of including video monitoring and electronic health records in future models [119].

Accelerometer and HR sensors were used in a small experiment (n = 2) to try to prevent challenging behaviors, using smartwatch technology to provide calming images and feedback when HR reached a certain threshold [120]. Something to note is that HR thresholding may capture episodes that caregivers and behavior specialists may not if there is no physical manifestation of stress [120], which is consistent with other studies using HR as an input mentioned above.

#### 5.1.4. Wearable Sensor Limitations

While the use of sensors to predict challenging behaviors is encouraging, not all individuals with profound ASD will tolerate a biosensor [104,114,121]—even biosensors sewn into clothing [116]—and sensors can be expensive to maintain in a large residential program. Finally, wearable devices only give minutes of notice [114] before challenging behavior, which may not be practical in a larger facility. Additionally, a sensor could detect potentially lower HRV, for example, but lower HRV may not always indicate stress and may give false positives [71].

### 5.2. Non-Wearable Sensors

#### 5.2.1. Bed Sensors

Behavior prediction currently uses several sleep sensor techniques, shown in Table 6, including smart beds [84]. In one smart sensor design, electromechanical film sensors (EMFis) were placed under the mattress to be unobtrusive and detect ballistocardiogram (BCG) signals for two individuals with profound ASD. Each individual had at least 200 nights of data recorded [84]. BCG signals were processed and used to detect motion and derive a measure of restlessness [84]. A thermal camera was also equipped to detect sleep time and sleep onset latency [84]. Using four, six, and eight prior nights of sleep worked best to predict behavior [84]. In a previous conference paper, Alivar and colleagues also tested the bidirectional relationship between sleep and behavior and found that more recent days of behavior could predict that night’s sleep features. An autoregressive model with both sleep and behavior features used to predict sleep performed even better [122]. Both SVMs and artificial neural networks (ANNs) were used to predict sleep and behavior; generally, ANNs performed better, although SVMs trained faster [84]. Alivar and colleagues suggested extending their work by applying a kernel SVM and including heart and respiratory rates as features [84].

#### 5.2.2. Computer Vision Approaches

Computer vision approaches, here defined as any approach that uses automated image analysis, are shown in Table 7. The majority of methods attempt to detect behaviors in the moment or predict them with short lead times, but a near-infrared (IR) camera setup showed decent levels of success over a longer timeframe. Kiarashi and colleagues recorded sleep features and motion at night for over two years from fourteen individuals with ASD and predicted challenging behaviors the next day [123]. Ensemble voting had the highest predictive success, with 74% accuracy [123]. The reduction in accuracy in comparison to other methods may be due to the longer lead time between observation and prediction in comparison to other methods.

Rajagopalan and Goecke created the self-stimulatory behavior dataset (SSBD), which included head-banging among other behaviors [137], with video and audio data taken from various different cameras and angles of 75 children’s facial expressions. Rajagopalan and Goecke applied a histogram approach to find the dominant motions in each video, then they used a bag-of-words approach to classify them into types of behavior, achieving a 10-fold cross-validation accuracy of 76.3% for behavior detection [124]. Liang et al. extracted features of self-stimulatory behaviors from the SSBD by applying an unsupervised temporal coherency deep network (TCDN) to videos of each behavior type, then they tested different classifiers at the frame and video level, obtaining 98.3% accuracy for both using quadratic discriminant analysis (QDA), although many classifiers had similar performances [125]. Another study focused on identifying three classes of behavior, head banging, arm flapping, and continuous spinning, achieving 83.56% validation accuracy using 3D-CNN and Skeleton Joint features [126]. Another recent approach used the SSBD to detect hand-flapping; Lakkapragada and coworkers used several feature extraction methods, finding MobileNetv2 to be the best, and put these extracted features into an LSTM network [127]. They achieved a test F1 score of 84%; however, this method presents privacy concerns, as the feature extraction uses facial features to detect movement [127]. The SSBD was also used with a long-term recurrent CNN, which achieved 96% accuracy [128]. This was the only detection method on the original SSBD that compared to the results found by Liang and colleagues. Kurian and Tripathi augmented the SSBD with videos available on YouTube to detect emotions in children with ASD [129]. They used a generative adversarial network (GAN) with a Wasserstein distance (WGAN). Among other emotional states, they could detect meltdown with high accuracy [129]. Wang and colleagues also created an expanded version of the SSBD, the Autism Restricted and Repetitive Behavior Dataset (ARRBD), using videos from other datasets [130]. Using a multimodal large language-based approach, the Multi-Model Synergistic Restricted and Repetitive Behavior Recognition method (MS-RRBR), on the ARRBD and text inputs, they were able to determine behavior with 94.94% accuracy [130]. Singh and colleagues also modified the SSBD, but instead of adding videos, they re-categorized the actions to include biting and slapping to allow for more nuanced SIB detection, as well as simulated alternative videotaping conditions by altering the videos to include artifacts [131]. The Long-Short Term Recurrent Convolutional Network (LSTRCN) was able to detect SIB with 92.62% accuracy [131].

Meltdowns were a common target for computer vision studies that did not use the SSBD or its derivatives. The first approach that focused on meltdowns was designed to detect gestures indicative of meltdown as they occurred and alert caregivers [83]. A recurrent convolutional neural network (RCNN) was trained on videos and images from online sources of gestures that indicated meltdowns. However, the model was only tested on five TD individuals, performing at approximately 92% [83]. Jarraya and colleagues initially used an RNN with three hidden layers on videos of 13 children with ASD recorded by a Kinect camera over a four-month period, achieving an accuracy of 85.8% [132]. In a later approach, Jarraya and colleagues recorded daily activities and meltdowns for 13 children with ASD over three months [133]. They again used geometric spatio-temporal deep features of facial expressions from videos to classify meltdowns and compare classification methods [133]. Of MLP, DT, SVM, KNN, and RF, RF performed the best, with approximately 91% accuracy [133]. The previous RNN was not compared.

Two studies have attempted to identify individual challenging behaviors under daily conditions. Manocha and Singh combined 3D-CNN and LSTM approaches to classify subtypes of SIB, aggression, and elopement for five individuals with ASD, achieving approximately 90% accuracy for each subtype [134]. A recent preprint used two cameras, one to the side and one above, in a classroom to identify and predict episodes of restricted, repetitive, disruptive, self-injurious, aggressive, elopement, and out-of-seat behaviors [135]. Combined multi-person pose estimation and multi-person tracking was used to follow individuals while preserving privacy [135]. A series of three methods, a Body Joint Attention model, a Temporal Convolution model, and a Person Attention model, were used to generate the features processed in a two-layer binary classification model [135]. The model could detect behaviors with a 77% F1 score, butit could only achieve a 53% F1 score when attempting to predict behaviors three minutes beforehand [135].

A recent pre-print by Zhao and colleagues attempted to detect and classify behaviors in a more replicable way by classifying behaviors in combined audio and video recordings using the 2nd edition of the Family Observation Schedule [136]. A CAV-MAE model was modified to improve its multiclass classification, resulting in the Audio–Visual FOS-II Encoding Neural Network. This network used segmented ten-second clips from handheld video of predesignated tasks in home settings, providing 0.8590 accuracy and 0.5936 F1 score and outperforming the other evaluated models when examined on individual classes [136].

### 5.3. Manually Recorded Approaches

Manually recorded data is uncommon in these approaches due to the time and resources needed to generate the data. However, several studies, shown in Table 8, have utilized various forms of it. Due to the logistical requirements, the majority of the data for these models comes from residential care facilities, biasing the population towards those with specific care needs and potentially limiting the model’s generalizability. However, the sample sizes are larger for these studies than the other approaches. A unique study by Cohen and colleagues focused on capturing data for a large number of participants in a residential care facility [138]. They recorded sleep data for 67 individuals and behavior data each day for five different behaviors, including SIB and aggression, as well as tantrums and property destruction. All were included in their challenging behavior class. Within that class, a significant predictive relationship between sleep and behavior was found for 81% of individuals [138]. Several sleep variables were used, including sleep duration, night awakenings, sleep onset and offset, and sleep regularity and efficiency [138]. They examined between 1 and 14 previous nights of data, finding that 8 nights was best for behavior prediction using a linear SVM [138].

Ferina et al. expanded on this work with data from a different residential facility by including GI and environmental data to predict SIB and aggression the next day from 1 day of prior data [139]. While fewer sleep features were available, the study used several GI features, pollen counts, moon phase, and weather data. The work identified a subgroup of 15–20% of individuals with an average next-day behavior prediction accuracy of ≥80% using an Adaptive Linear Neuron (ADALINE) model with direct kernel transformation [139].

A recent paper included autoregressive models—behavior from the previous 7 or 14 nights—to predict aggression, SIB, elopement, and medical events the next day for the same residential care program that Ferina et al. worked with [140]. Kiarashi and colleagues created a binary presence/absence feature vector across the most prevalent high-level behavior categories and seizure occurrence, then they used this as a sliding window for 7 or 14 days to predict behavior the next day using a CNN. The 7-day model provided better predictions, with 78.4% accuracy for aggression, 80.68% for SIB, and 85.43% for elopement. The feature analysis showed that future aggression was best predicted by past aggression, then disruptive behavior and elopement. In contrast, SIB was best predicted by disruptive and other behaviors, and elopement by disruptive behavior. The temporal relation and co-occurrence of these challenging behaviors is an intriguing avenue to explore in future works.

Another paper examined AI’s ability to determine ten individual behaviors from descriptions of events using the Text-based Early Autism Spectrum Disorder Detection Dataset for Toddlers (TASD). The Bidirectional Encoder Representations from Transformers (BERT) used natural language processing to achieve an average performance of 88% [141].

## 6. Challenging Behavior Intervention and Reduction

The next step for behavior prediction is to integrate predictions into interventions to prevent harm. Some treatments and intervention strategies will benefit more from behavior prediction than others. Interventions should also be examined as links to potential features for behavior prediction. While behavior interventions as moderators of future behavior are beyond the scope of this review, they are of interest for future work. An exhaustive review of treatment and intervention strategies is beyond the scope of this review; instead, a brief overview is provided, and possible avenues for integration with behavior prediction are noted in the text and summarized in Table 9.

### 6.1. Assessment

#### 6.1.1. Functional Behavior Assessment

FBA is standard practice to determine what patterns and environmental factors are causing, encouraging, or reinforcing challenging behaviors [21,142]. FBA has several subsets, including indirect and functional analysis. Indirect FBA assess questionnaires and interviews with individuals who observe the behavior rather than the individual directly. Functional analysis instead observes the individual’s responses during a series of tests where environmental variables are controlled or manipulated [21]. While variable between individuals, certain aspects of these assessments may lend themselves well to incorporation into a behavior prediction model. FBA is not necessarily able to determine a challenging behavior’s origin [21], but it can determine which interventions may be the most effective for an individual [21,142] and increase the likelihood of a positive outcome [142,143].

#### 6.1.2. Stimulus Assessment

The majority of those with ASD, over 90% in one study, have an atypical response to sensory stimuli, which has been theorized to be linked to challenging behaviors [144]. There are two primary types of stimulus assessments, which provide complimentary information about an individual’s responses: stimulus preference assessments and competing stimulus assessments. The first determines what stimuli are “preferred” or pleasant, and the second attempts to determine what stimulus is correlated with the challenging behavior and is, presumably, an irritant. This knowledge can be used to complement other treatment methodologies [21].

### 6.2. Pharmacological

Behavior prediction would presumably primarily be useful for pharmacological interventions in the case of “as-needed” medications or ones taken as or immediately before symptoms start, as opposed to regular regimes, which are currently standard. Pharmacological interventions typically target specific behaviors [39,144]; therefore, the medications used vary depending on the target behavior or behaviors [145]. Certain challenging behaviors, such as aggression and SIBs, appear to be more commonly treated with medication [144,145,146] than others, such as elopement. Medications and their dosing schedules also vary depending on COCs [145]. Commonly used medications include second-generation or atypical antipsychotics [39,144,145,146], mood stabilizers [39,144,145], and antiepileptic medications [39,144,145], among others [39]. A significant portion of medications used are off-label [144] or are studied primarily in children [145], who can have different side effects from adults [39]. Additionally, side effects, most frequently weight gain, are common for these medications [39,144]. An uncommon, but concerning, side effect of ASD medications is aggression, which can either be caused directly or indirectly by side effects of fatigue, sleep issues, nausea, and other symptoms that have been associated with aggression [12,38,48].

### 6.3. Non-Pharmacological

There is a wide range of potential applications of behavioral prediction for non-pharmacological interventions; examples include reducing the timeframes that burdensome preventative measures have to be in place, removing an individual with ASD from a stimulating environment for intervention before an issue occurs, and improving the targeting of behavioral intervention plans. In part, implementation of behavioral prediction will depend on its precision and lead time before the onset of the challenging behavior.

Applied behavior analysis (ABA) and its derivatives are the most prominent intervention approaches for challenging behaviors for individuals with ASD [21,142]. Traditionally, ABA focused on retraining behaviors [144] using antecedent and consequence-focused therapy [142]. However, its methodology has shifted to focus on underlying emotional regulation and mental health [142,147]. A notable derivative of ABA is the emerging discipline of positive behavior support (PBS) [147]. The most prominent difference between ABA and PBS is that PBS is defined by its refusal to use aversive or restrictive techniques [147,148] rather than incorporating these values later in the field’s development [65,142,147].

There are numerous other approaches for challenging behaviors, primarily behavior intervention or environmental manipulation. Many behavioral intervention approaches are informed by ABA, despite not being as directly derived as PBS [149,150]. For example, skill-based treatment is a derivative of functional communication training, which seeks to define variables associated with behavior and then systematically alter the variables to teach social and emotional regulation skills [149]. There have also been technological advances in behavioral interventions, such as the use of social robots [151,152]. Robots have some advantages in that they provide a predictable, simplified interaction [152] that can be repeated to allow those with ASD to learn a single skill in as much time as they need, rather than dealing with variable and multifaceted social interactions with another person [151]. Robots can then gradually increase the complexity if needed [151]. A review of 9 small studies, the largest including 36 children, found that social robots can reduce challenging behaviors [152]. However, many robots do not focus on challenging behaviors directly, instead focusing more generally on social and emotional skills. A meta-analysis of small studies, the largest of which looked at 30 boys, found that robots could improve social and communication skills, but the 2 studies that focused on emotional skills did not show an improvement [151]. While robots do not currently integrate behavioral prediction software, they may be in a unique position to benefit if their responses can be adjusted based on the predictions. Some robots are progressing in this direction by working to integrate emotion [153] and aggression [81] recognition software. Environmental modifications are used in certain situations. In a regular environment that is expected to provoke challenging behavior, e.g., a classroom, mixed results have been obtained by reducing stimuli or adding a pleasant stimulus [143]. Certain behaviors, like elopement, can be managed with environmental modifications, e.g., physical barriers or tracking devices [17]. A current limitation of tracking devices is the discomfort and inconvenience associated [154]. Having advanced notice of when they would be necessary may limit the amount of time they would be required and make them more appealing.

While the majority of non-pharmacological interventions fall into the categories of behavioral intervention or environmental manipulation, other avenues are being explored. For example, there have been several small studies on deep brain stimulation (DBS) to treat what was defined as extreme behaviors, primarily aggression and SIBs [146,155]. DBS attempts to alter and regulate brain function using electrical stimulation, though what aspects of brain function should be altered to reduce aggression and how to position the electrodes to achieve that are still being explored [146,155]. However, due to the invasive nature of DBS, it is not recommended as a first-line treatment [155].

### 6.4. Transdisciplinary

Although the majority of research examines individual methods or individual methods in conjunction with an assessment [142,143], it is increasingly common to use a combination of interventions [21]. Individuals with ASD, especially those with multiple challenging behaviors or COCs, may benefit from cross-disciplinary teams assessing and treating the same issue from different perspectives [21].

## 7. Limitations of the Field

Challenging behavior may be caused by multiple physiological and psychological reasons. While efforts have been made to address this, there has yet to be a large study examining both aspects together. Additionally, behavior is highly individual, and population models—although useful when there are not resources to devote to one individual at a time—may not provide the underlying reasons or a complete view of the reasons for the challenging behavior. Determining the reason for a behavior can also be difficult if the individual is minimally or non-verbal, so the typical approach is to use interventions to determine what reduces episodes of the challenging behavior. However, these studies are often performed in controlled settings with small sample sizes, so determining how this translates to the individual’s daily life and the population as a whole is challenging.

## 8. Conclusions and Opportunities for Future Work

Overall, there exists a wealth of promising work on using biosensors to predict challenging behaviors, but this research has not yet been clinically translated. Furthermore, there are opportunities to combine sensor data with psychological data that have not been previously explored. Sensors are generally popular tools to detect behavior, although not all individuals in the ASD population will tolerate biosensors, which has been shown in multiple studies. A current limitation of most biosensor work is that it gives very little advance notice before the onset of a challenging behavior, so it is only applicable when the individual can easily and quickly be removed from a problematic situation. One interesting opportunity for expansion is to use prior sleep data from a wearable biosensor to predict behavior outcomes the next morning. This approach has the potential to provide caregivers with reliable, objectively collected data, and also to enable them to predict behaviors in advance, unlike current wearables. Such an approach could also expand upon the data taken by bed sensors—movement only—to include skin temperature and HRV and would not require a custom bed setup to implement. Studies using these wearable sensors report inconsistent results on which model type is best for behavior prediction, and many of these studies are relatively small, with the exception of ones performed in residential facilities. One observation that is consistent across studies is that individual models provide better results than population models, necessitating a personalized medicine approach to challenging behavior treatment and prediction.

If sensors cannot be tolerated or are too costly, there is also promising work by researchers using prior manually recorded data, computer vision, and off-body sensors to predict challenging behaviors. Computer vision approaches are able to detect movement while it is happening and predict behavior from movements during sleep. Manually recorded approaches are less studied and come with caveats that manually recorded data may be impossible for at-home caregivers to consistently record, but this method of behavior prediction is promising for residential facilities.

Regardless of the approach best suited to the individual and setting, all reviewed approaches demonstrate the potential of using an individual’s physiological history and information to predict their future behavior. The current base of work suggests several paths for future work to improve prediction accuracy and the ability to differentiate between behavior types, reduce the intrusion of the sensors, and address privacy concerns. However, future systematic and meta-analyses are needed to better analyze the results of each approach to more clearly evaluate their potential. Future development and eventual clinical translation has the potential to personalize and improve the precision and efficacy of treatment, helping to improve quality of life of individuals with ASD and their caregivers.

## Figures and Tables

**Table 1:** Challenging behavior definitions and examples.

Behavior Type	Definition	Examples	Prevalence in Those with ASD	Source
Aggression	Aggressive acts towards other people	Biting, head-butting, threatening, scratching	51.7–56%	[5,15]
Self-injurious behavior (SIB)	Acts that harm the individual	Pulling hair, banging head, poking eye	28.0–42%	[5,15,16]
Elopement	Leaving an area without permission	Running away, bolting, leaving the room	32.7–49.1%	[17,18,19]
Disruptive behavior	Any act that could be considered disruptive or inappropriate for the situation	Yelling, tantrums, inappropriate sexual behaviors	21.0–36%	[13,20]

**Table 2:** COC groupings, their reported prevalence, and how they can impact behavior for individuals with ASD.

Type of COC	Prevalence in Individuals with ASD	Prevalence Citation	Association with Challenging Behavior
GI	9–91%	[9]	Increased aggression and SIB, depending on GI symptoms
Epilepsy	11.5–30%	[12,26]	Increased SIB
Sleep	13–80%	[9,27]	Increased SIB and aggression, depending on type of sleep disorder
Allergies and Immune	11.25%	[28]	Mixed associations

**Table 3:** Studies that attempt to detect or predict emotion or behavior in individuals with ASD using physiological signals. NS: validation method not stated, CV: cross validation, LOOCV: leave-one-out CV.

Study	Sample	Signals Used	Model Used	Results
Conn et al. (2008) [86]	(n = 6) Children with ASD	ECG, EDA, EMG, reports of arousal level	SVM [LOOCV]	Prediction accuracy: 79.5% (anxiety), 85% (liking), 84.3% (engagement)
Sarabadani et al. (2018) [87]	(n = 15) Children with ASD	ECG, skin conductance, skin temperature, and respiration measures	Majority vote from 5 classifiers [CV, 75:25 train:test]	Positive or negative high-arousal state detection: 78.1% average accuracy
Van Laarhoven et al. (2021) [88]	(n = 5) Young adults with ASD	Respiration patterns	Spire Stone app [NS]	Significant relationship: 2/5
Ferguson et al. (2019) [89]	(n = 8) Adolescents with ASD (age 13–20)	EDA	Statistical analysis [NS]	Anticipatory rise in EDA: 60%, EDA returned to median baseline levels: 45%
Freeman et al. (1999) [90]	(n = 2) Adult men with developmental disabilities	ECG sensors to detect HR	HR scale [NS]	HR increased after challenging behaviors
Freeman et al. (2003) [91]	(n = 1) Dataset of individual with intellectual disability	HR, observations	LERS [NS]	Set of rules to predict under what conditions certain types of behavior were likely to occur
Hoch et al. (2010) [92]	(n = 1) Male child with ASD	HR	General transformation approach to auto-regressive integrated moving average [Baseline found separately]	Significant associations between high or low arousal activities
Lydon et al. (2023) [93]	(n = 3) Male individuals with ASD and ID	HR (band around the torso)	*T*-test [NS]	Significant SIB difference for 1 participant
Nuske et al. (2019) [94]	(n = 13) Preschoolers with ASD	Baseline-corrected HR	Conditional LR [Baseline found separately]	Predict challenging behavior: 0.72 AUROC
Masino et al. (2019) [95]	(n = 22) Children with ASD	HR and beat-to-beat parameters	RBF kernel SVM model [LOOCV]	Classify stressed and relaxed states: 91% AUC
Koumpouros (2021) [96]	(n = 20) Children with high-functioning ASD	HR	HR threshold [NS]	≥1/3 were false or unidentified alarms
Khullar et al. (2021) [97]	(n = 10) Children with ASD, (n = 5) TD children	HR, galvanic skin response, and skin temperature	Hybrid CNN–LSTM network [10 fold CV]	Meltdown prediction: 96% accuracy
Bagirathan et al. (2021) [98]	(n = 6) Children with ASD (ages 5–11)	ECG	KNN, SVM, and ensemble classifier [NS]	Like or dislike detection: 81% (HRV, ensemble)

**Table 4:** Studies that attempted to detect or predict emotion or behavior in individuals with ASD using movement sensors. NS: validation method not stated, CV: cross validation, LOSOCV: leave-one-subject-out CV, LOTOCV: leave-one-trial-out CV.

Study	Sample	Signals Used	Model Used	Results
Albinali et al. (2009) [99,100]	(n = 6) Adolescents with ASD (age 13–20)	Accelerometer	C4.5 classifier [10-fold CV]	SMM detection accuracy: 82.5–96.4% (laboratory), 85.9–93.7% (classroom)
Goodwin et al. (2014) [101]	(n = 6) Individuals with ASD	Wrist and torso-worn accelerometers	SVM [10 fold CV]	SMM detection: 81.2–99.1% accuracy
Rad et al. (2016) [102]	(n = 6) Children with ASD	Accelerometer	CNN and transfer learning [LOSOCV]	SMM detection: 0.75 (time 1), 0.48 (time 2) F1 score
Siddiqui et al. (2021) [103]	(n = 10) Children with ASD	Wrist sensors (accelerometer, gyroscope)	RF, NN, KNN [10-fold CV and LOSOCV]	91% gesture recognition
Plötz et al. (2012) [104]	(n = 1) Child with ASD	Wrist and ankle accelerometer	SVM [10-fold CV]	69.7% accuracy to detect challenging behaviors
Min et al. (2009) [105]	(n = 2) Children with ASD	Wrist and body sensor, back sensor	Accelerometer [NS]	Average classification accuracy: 95.5% rocking (back), 80.5% flapping (back), 86.5% flapping (wrist)
Min and Tewfik (2010) [106]	(n = 4) Children with ASD	Accelerometer	Autoregressive model [separate training and test sets]	92.7% average self-stimulating prediction accuracy
Min (2017) [107]	(n = 4) Children with ASD	Accelerometer and video	HMM [LOTOCV]	91.5% self-stimulatory behavioral pattern detection
Westeyn et al. (2005) [80]	(n = 1) NT adult mimicking behaviors	Accelerometer wrist, waist, and ankle sensor	HMM, isolated and continuous recognition [LOOCV]	Self-stimulatory behavior detection: 90.5% accuracy (isolated), 92.86% accuracy (continuous)
Cantin-Garside et al. (2020) [108]	(n = 11) Children with ASD (age 5–14)	Variable numbers of accelerometers	Multilevel LR [80:20 train:test]	74.7% SIB prediction accuracy
Cantin-Garside et al. (2020) [109]	(n = 11) Children with ASD (age 5–14)	Variable numbers of accelerometers	KNN [6:2:2 train:validate:test]	SIB detection: 93.0%
Alhaddad et al. (2019) [81]	(n = 5) NT adults (training) and (n = 4) NT children (validation)	Accelerometers in toys	RNN [separate training and testing sets]	80% F1 score (classify hit, shake, throw, pickup, drop, and idle)

**Table 5:** Studies that attempt to detect or predict emotion or behavior in individuals with ASD using combined sets of signals. NS: validation method not stated, CV: cross validation, LOOCV: leave-one-out CV.

Study	Sample	Signals Used	Model Used	Results
Goodwin et al. (2019) [111]	(n = 20) Children with ASD	Wrist sensor (EDA, IBI, BVP and accelerometer)	Ridge-regularized LR [5 fold CV]	Aggression prediction AUC: 0.84 (individual), 0.71 (population)
Alban et al. (2023) [112]	(n = 5) Children with ASD	Acceleration, EDA, skin temperature, HR, and BVP	XGBoost [70:30 train:test]	Challenging behavior detection: 99% accuracy
Imbiriba et al. (2020) [113]	(n = 20) Children with ASD	EDA, BVP, accelerometer	PCA with RBF kernel SVM [4 and 6-fold CV]	Aggression detection: 98% accuracy
Imbiriba et al. (2023) [114]	(n = 70) Children with ASD	Wrist sensor (EDA, BVP, accelerometer) augmented by behavioral data	LR [5 fold CV]	Predict aggression 3 min prior: 80% AUROC
Al Banna et al. (2020) [82]	NT facial expression dataset	Camera, RFID toys, wearable (accelerometer, HR, gyroscope, magnetometer, GPS, pedometer, temperature)	CNN [separate training and testing sets]	CNN emotion detection in images: 78.56% accuracy
Manu et al. (2024) [115]	(n = 5) Male children with ASD	Acceleration, EDA, HR, BVP, and temperature	XGBoost [NS]	Challenging behavior detection: 91% (accuracy), 88% (F1 score)
Zheng et al. (2021) [116]	(n = 7) Children with ASD	Microsoft Kinect, sensor hoodie, and wristband (head rotations, BVP, EDA, facial expressions, acceleration, and temperature)	RF, NN (population), RF (individual) [4 fold CV, LOOCV]	Challenging behavior prediction accuracy: 98.51% (individual), 82.36% (population)
Zwilling et al. (2022) [117]	Adults with ASD (ages 20–40)	Clothing sensors (ECG)	HR threshold and LSTM [NS]	Results not reported yet
Gonçalves et al. (2012) [118]	(n = 4) Children with ASD	Accelerometer watch, Microsoft Kinect	Statistical methods (accelerometer), gesture recognition (Kinect) [CV]	Hand-flapping stereotypy classification accuracy: 76% (accelerometer), 51% (Kinect)
Rad et al. (2025) [119]	(n = 11) Adolescent males with ASD (ages 10–20)	EDA, three-lead accelerometer, and skin temperature monitoring	LSTM [10 fold CV]	Challenging behavior detection AUROC: 0.710 (accelerometer), 0.524 (EDA), 0.629 (skin temperature)
Torrado et al. (2017) [120]	(n = 2) Male children with ASD (age 10)	HR and accelerometer	HR threshold [baseline found separately]	System was effective for intervention

**Table 6:** Studies that attempt to detect or predict emotion or behavior in individuals with ASD using bed sensors. NS: validation method not stated, CV: cross validation.

Study	Sample	Signals Used	Model Used	Results
Alivar et al. (2019) [122]	(n = 2) Individuals with profound ASD	EMFis to detect BCG	SVM and ANN [separate training and testing sets]	ANN using sleep onset latency: 85% accuracy, 79% F1 score
Alivar et al. (2020) [84]	(n = 2) Male children with ID	EMFis to detect BCG	SVM [70:30 train:test] and ANN [70:15:15 train:validate:test]	Collective behavioral prediction: ≥78%

**Table 7:** Studies that attempt to detect or predict emotion or behavior in individuals with ASD using computer vision. NS: validation method not stated, CV: cross validation.

Study	Sample	Signals Used	Model Used	Results
Kiarashi et al. (2024) [123]	(n = 14) Individuals with ASD	IR camera	Ensemble voting model [80:20 train:test]	Challenging behavior prediction: 74% accuracy
Patnam et al. (2017) [83]	(n = 5) TD individuals	Camera	RCNN [15–25% validation]	Meltdown gesture detection: 92% accuracy
Rajagopalan and Goecke (2014) [124]	SSBD	Video and audio files	Histogram, bag of words [5 and 10-fold CV]	Behavior detection: 76.3% accuracy
Liang et al. (2021) [125]	SSBD	Video and audio files	TDCN and SVM, TDCN and QDA [5-fold CV]	Behavior detection: 98.3%
Shanmughapriya and Poojashree (2022) [126]	SSBD	Video and audio files	3D-CNN and Skeleton Joint features [80:20 train:test]	Accuracy: 83.56% (dataset validation), 65% (cross data)
Lakkapragada et al. (2022) [127]	SSBD	Video and audio files	MobileNetv2 and LSTM [5 fold CV]	Hand-flapping detection: 84% F1 score
Alkahtani et al. (2023) [128]	SSBD	Video and audio files	Long-term recurrent CNN [80:20 train:test]	Behavior detection: 96% accuracy
Kurian and Tripathi (2024) [129]	SSBD and videos of children with ASD	Video and audio files	GAN with WGAN [32:82 samples train:test]	Behavior detection accuracy: 93.75% (train), 88.25% (test)
Wang et al. (2025) [130]	Video and audio files and text input	ARRBD and text inputs	MS-RRBR [7:2:1 train:validate:test]	Behavior detection accuracy: 94.94%
Singh et al. (2025) [131]	Enhanced SSBD	Video and audio files	LSTRCN [80:20 train:test]	SIB detection accuracy: 92.62%
Jarraya et al. (2020) [132]	(n = 13) Children with ASD	Microsoft Kinect	RNN with 3 hidden layers [10 fold CV]	Meltdown detection: 85.8% accuracy
Jarraya et al. (2021) [133]	(n = 13) Children with ASD	Camera	RF [70:30 train:test]	Meltdown detection: 91% accuracy
Manocha and Singh (2023) [134]	(n = 5) Individuals with ASD	Camera	Combined 3D CNN and LSTM [80:20 train:test]	90% detection accuracy (SIB, aggression, and elopement)
Das et al. (2024) [135]	(n = 6) Children with ASD	Cameras (side and above)	Two-layer binary classification model on generated features [5-fold CV, 50:20:30 train:validate:test]	F1 score: 77% (behavior detection), 53% (3 min prior prediction)
Zhao et al. (2025) [136]	(n = 83) Children with ASD	Audio and video	Modified CAV-MAE [6173:1867 videos train:test]	FOS-II behavior classification: 0.8590 accuracy, 0.5936 F1 score

**Table 8:** Studies that attempt to detect or predict emotion or behavior in individuals with ASD using manually recorded data. NS: validation method not stated, CV: cross validation.

Study	Sample	Signals Used	Model Used	Results
Cohen et al. (2018) [138]	(n = 67) Individuals with low-functioning ASD	Sleep duration, night awakenings, sleep onset and offset, and sleep regularity and efficiency	Linear SVM [10 fold CV]	Significant predictive relationship between sleep and behavior for 81% of individuals
Ferina et al. (2023) [139]	(n = 80) Individuals with ASD (age < 19)	Behavior, GI, sleep, and environmental data	Direct kernel ADALINE [85:15 train:test]	15–20% of the cohort reached 80% prediction accuracy
Kiarashi et al. (2024) [140]	(n = 353) Individuals with ASD	Presence/absence of behavior and seizure data	Autoregressive models [80:20 train:test]	Prediction accuracy: 78.4% (aggression), 80.68% (SIB), 85.43% (elopement)
Monalin and Rubini (2024) [141]	TASD	Written observations	BERT [80:20 train:test]	Average behavior detection: 88%

**Table 9:** An overview of potential ways that behavior prediction may be used in conjunction with current intervention protocols.

Method	Description	Examples	Potential Behavior Prediction Application
Assessment	Observations or tests to determine influences and impetuses of challenging behavior	FBA and stimulus assessment	Aspects of assessments may be incorporated into a behavior prediction model
Pharmacological	Medication-based interventions	Second-generation antipsychotics, mood stabilizers	Could allow for or improve the timing of as-needed medications
Non-Pharmacological	Interventions which do not use medications	ABA, music therapy, electronic tracking devices	Improve timing of interventions
Transdisciplinary	Intervention approaches that combine two or more of the above methods	Skill-based treatment combined with antipsychotics, ABA with music therapy	Improved timing of interventions, especially to appropriately pair or stagger approaches

## Data Availability

Not applicable.

## Abbreviations

The following abbreviations are used in this manuscript:

ASD	autism spectrum disorder
SIB	self-injurious behavior
COC	co-occurring condition
GI	gastrointestinal
SMM	stereotypical motor movements
ADDM	Autism and Developmental Disabilities Monitoring
CDC	Center for Disease Control
ID	intellectual disability
ODD	oppositional defiant disorder
TD	typically developing
GERD	gastroesophageal reflux disease
NT	neurotypical
FBA	functional behavior analysis
ECG	electrocardiogram
EDA	electrodermal analysis
EMG	electromyography
SVM	support vector machine
NS	validation method not stated
CV	cross validation
LOOCV	leave-one-out cross validation
HR	heart rate
LERS	learning from examples using rough sets
AUROC	area under the receiver operator curve
HRV	heart rate variability
AUC	Area under the curve
RBF	radial basis function
CNN	convolutional neural network
LSTM	long short-term memory
DT	decision tree
RF	random forest
NN	neural network
KNN	k-nearest neighbors
LOSOCV	leave-one-subject-out cross validation
LOTOCV	leave-one-trial-out CV
HMM	hidden Markov model
LASSO	least absolute shrinkage and selection operator
PCA	principal component analysis
LR	logistic regression
RNN	recurrent neural network
IBI	inter-beat interval
BVP	blood volume pulse
MLP	multilayer perceptron
XGBoost	extreme gradient boosting
RFID	radio frequency identification
EMFis	electromechanical film sensors
BCG	ballistocardiogram
ANN	artificial neural networks
IR	infrared
RCNN	recurrent convolutional neural network
SSBD	self-stimulatory behavior dataset
TCDN	temporal coherency deep network
QDA	quadratic discriminant analysis
GAN	generative adversarial network
WGAN	Wasserstein distance
ARRBD	Autism Restricted and Repetitive Behavior Dataset
MS-RRBR	Multi-Model Synergistic Restricted and Repetitive Behavior Recognition method
LSTRCN	long short-term recurrent convolutional network
ADALINE	adaptive linear neuron
TASD	Text-based Early Autism Spectrum Disorder Detection Dataset for Toddlers
BERT	bidirectional encoder representations from transformers
ABA	applied behavior analysis
PBS	positive behavior support
DBS	deep brain stimulation
